# The usefulness of a clinical 'scorecard' in managing patients with sore throat in general practice

**DOI:** 10.1186/1447-056X-9-9

**Published:** 2010-07-29

**Authors:** Tony MO Bakare, Peter Schattner

**Affiliations:** 1 Department of General Practice, School of Primary Health Care, Bldg 1, 270 Ferntree Gully Rd, Notting Hill, Melbourne, Victoria 3168, Australia

## Abstract

**Background:**

Objective: To evaluate the usefulness of a clinical scorecard in managing sore throat in general practice.

Design: Validation study of scorecard for sore throat with a throat swab culture used as the 'gold standard'.

Setting: A solo family practice in rural New South Wales, Australia

Participants: Patients attending with sore throat.

**Methods:**

Patients from the age of 5 years and above presenting with the main symptom of a sore throat, and who have not had any antibiotic treatment in the previous two weeks, were invited to participate in the study. The doctor completed a scorecard for each patient participating and took a throat swab for culture. Adult patients (> 16 yrs) were asked to complete a patient satisfaction questionnaire, while guardians accompanying children (5 yr to < 16 yrs old) were asked to complete a similar, guardian questionnaire.

Main outcome measures:

1. Ability of a new scorecard to differentiate between bacterial and non-bacterial sore throat.

2. Patients' trust in the scorecard.

**Results:**

The scorecard has a sensitivity of 93.33%, a specificity of 63.16%, a positive predictive value of 50% and a negative predictive value of 96%. The sensitivity is better than other sore throat scorecards that have been published but with a slightly lower specificity.

There was a high level of patient trust in the scorecard was (85.8% agreement). Patients also trusted their doctor's judgement based on the scorecard (90.6% agreement).

**Conclusions:**

As the scorecard has a high sensitivity but only a moderate specificity, this means that it is more reliable for negative results, i.e. when the result suggests a viral infection. When the result favours a bacterial sore throat, then a high sensitivity can mean that there are a number of false positives. GPs can be confident in withholding antibiotics when the scorecard indicates a viral infection.

## Introduction

The management of sore throat in general practice is traditionally based on the doctor's clinical judgment and empirical treatment. However, as the rate of prescribing remains quite high for a condition mostly due to viral causes, distinguishing between non-bacterial and bacterial causes of sore throat is still important [1].

Clinical scoring systems have been developed to help recognise bacterial or non-bacterial sore throats, e.g. Centor's and Breese's criteria, which are based on 4 and 9 items respectively, and which only use clinical variables (see Table 1). However, there are several shortcomings to existing systems such as limitations in their sensitivity (Breese 68%; Centor 65% to 83%) and their specificity (Breese 85%; Centor 67%-91%) [2]. An accurate scorecard will remain valuable whenever alternative techniques for identifying bacterial or non bacterial causes, such as rapid antigen testing and throat swab culture, are unaffordable, unavailable or impractical.

**Table 1:** Comparison of new scorecard with other scoring systems used in diagnosing group A streptococcal (GAS) pharyngitis

Study author (year)	Features included in final algorithm	Age group (years)	Sample size	Sensitivity (%)	Specificity (%)	Positive predictive value(%)
The current scorecard	Ten criteria (See Figure 1)	5 yrs to 99 yrs	106	93.3	63.2	50

Breese‡ (1977)_15_	Season (late winter or early spring) Age (5-10 years) Elevated white blood cell count Temperature > 38°C Sore throat Absence of cough Headache Pharyngeal erythema or oedema or exudates Tender or enlarged cervical lymph nodes	Children	670	68	85	84

Centor† (1981)_16_	History of fever Tonsillar exudates Tender and enlarged anterior cervical lymph nodes Absence of cough	> 15	234	65-83	67-91	56

**Figure 1:**
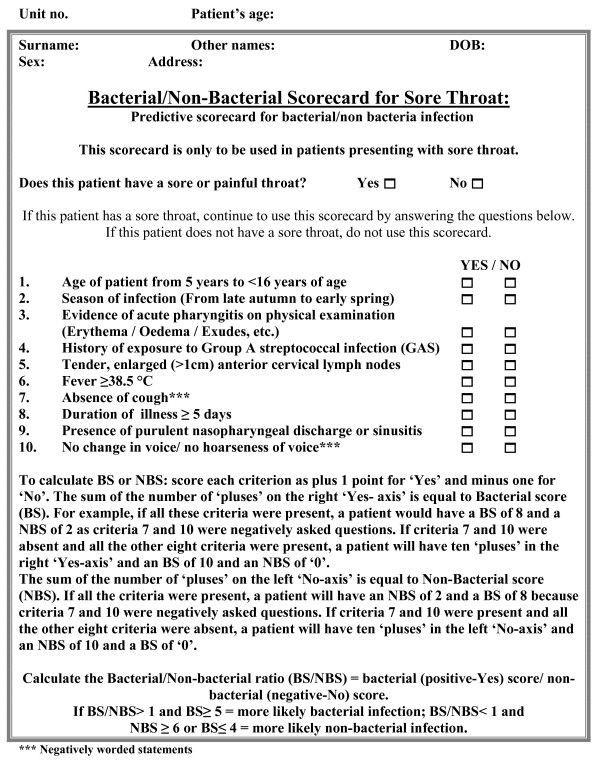
**The study scorecard**.

This study sought to ascertain whether a new scorecard shows benefits over other ones published in the medical literature. For example, the widely used Centor scorecard has less than optimal sensitivity and specificity. When tested in an urban emergency department using the four criteria, throat culture was positive in 56% of these patients and in patients meeting three criteria, the positive predictive value of a positive culture was only 30% to 34% [3].

The new scorecard was developed from ten criteria which included 4 from Centor (i.e. tonsilar exudates; tender anterior cervical adenopathy; fever by history; and absence of cough). One criterion was taken from Breese's study [4] (season of illness - late winter or early spring), and one from Wald's study [5] (age 5-15 years).

The four remaining criteria were selected by the investigator as they seemed relevant to sore throat and were considered useful in increasing the accuracy of the scorecard, based on the author's experience. These criteria included:

• History of exposure to group A streptococcal sore throat infection [6].

• Purulent nasopharyngeal discharge associated with sore throat [7].

• Duration of illness > 5 days; longer duration of symptoms or illness may indicate a Strep A sore throat [8].

• History of hoarseness or changes in voice - this symptom has been associated with a high negative predictive value [9,10].

The investigator was of the view that by including additional clinical features to the 4 Centor's criteria, the accuracy of a scoring system could be improved. The aim of this study was therefore to evaluate the validity of a newly developed clinical scorecard in managing sore throat and also to determine the acceptability of this scorecard by patients and carers. While an instrument may be successfully validated, it may not necessarily appeal to the subjects for which it was intended, in this case, the patients and carers. Hence, the importance of also studying the acceptability of the scorecard.

## Methods

### Setting

The study was conducted by the investigator, a solo family physician working in Cobar, a rural town in New South Wales, Australia, with a population of close to six thousand people. The investigator worked full time in his medical practice which was open for 9 -10 hours daily, six days a week. There were on average 30 to 50 patients per day, with over two hundred per week. The research took place at the investigator's practice between April and December 2006 following ethics committee approval from Monash University.

### Scorecard development

The Centor criteria have provided the most widely used and accepted scorecard in America and in many other countries. The proposed new scorecard of ten factors, therefore, contains Centor's four factors and six additional factors as explained in the previous section. The final version of the scorecard is presented in Figure 1.

### Questionnaire development

A patient/guardian perception questionnaire on sore throat was developed for this study using a five point Likert scale (5 Strongly agree, 4 Agree, 3 Neutral, 4 Disagree and 1 Strongly disagree). This questionnaire examined attitudes to and beliefs about the use of a scorecard and also collected additional information such as the severity of symptoms of the current sore throat.

### Subject recruitment

Patients were made aware of the study via a poster placed in the waiting room and at the reception area. Patients from the age of 5 years and above presenting predominantly with a sore throat and who had not had any antibiotic treatment in the previous two weeks were sequentially invited to participate. If they or their guardian agreed, one or the other then signed a consent form.

### Procedure

A throat swab was taken from all patients participating in the sore throat study by rubbing vigorously against each tonsil as recommended by Brien and Bass [11]. After taking the swab, the doctor completed a scorecard for each patient participating in the study. Adult patients (>16 yrs) were asked to complete a patient questionnaire, while guardians accompanying children (5 yr to <16 yrs old) were asked to complete a similar, guardian questionnaire. Patients either completed the questionnaire while still in the consulting room or in the waiting room. This was then placed in a secure box in the reception area or handed back to the doctor by the receptionist.

### Handling of throat swab

The throat swab was transported in an individually covered Stewart's transport medium to the regional pathology service in Dubbo, New South Wales, Australia, for microscopy, culture and sensitivity. The throat swabs were forwarded within 24 hours of collection.

Cultures showing any group A beta haemolytic Streptococci, Streptococci groups B, C and G, and staphylococci were considered positive. Cultures showing mixed respiratory flora, Candida and non-specific growths were considered negative. Undertaking viral studies was not feasible in this setting and so the absence of bacterial growth was assumed to reflect a viral infection or other minor causes such as allergy.

### Data analysis

Patients were assigned to one of two groups based on the results of the scorecard:

• Bacterial group A: patients who had a bacterial score of ≥ 5 and a non-bacterial score of ≤ 4.

• Non-bacterial group B: patients with a non-bacterial score of ≥ 6 and a bacteria score of ≤ 4 on the scorecard.

The data obtained from the scorecard, the throat swab pathology reports from the laboratory and the questionnaires were checked and then tabulated using the Microsoft Excel program. They were subjected to a recheck and analysis by a statistician. The sensitivity, specificity, predictive values and the accuracy of the scorecard were determined by comparing the scorecard findings with the gold standard of the throat swab. Data from the completed patient and guardian questionnaires were analysed using histograms to plot the frequencies of responses to the questionnaire against the 5 Likert scale responses. The Pearson's product moment test was used to confirm that the correlation between the scorecard and the throat swab results were within an acceptable range. The analysis was repeated using the non-parametric Spearman's rho test. Cronbach's alpha was used to verify that the internal consistency of the guardian and adult questionnaires was within an acceptable range.

## Results

### Subject participation

Over a thousand patients were seen between April and December 2006, out of which one hundred and eighteen patients presented with sore throat. All of these were approached to enter the study, but 12 patients were excluded either because they had refused a throat swab or refused to complete a questionnaire.

Participants in the study included twenty-four children under the age of 16 years who were accompanied by parents or guardians (22 mothers, 1 father and 1 grandmother), with fourteen of the children being male and with a mean age was 8.71 years (standard deviation 3.76, range 5 to <16 yrs). There were 82 adult patients, with 30 of them being male. The mean age in the adult group was 34.85 (standard deviation 15.25, range 15 to 81).

### Child group

In the child group, 7 patients had a true positive result for bacterial infection (i.e. scorecard and microbiological culture in agreement) and none had a false negative result, i.e. a sensitivity or true positive rate of 7/7, or 100% (Table 2). Further, 9 patients had a false positive result for non-bacterial infection (positive on scorecard and negative on microbiological culture) and 8 patients had a true negative (negative on scorecard and negative microbiological culture). This means that the specificity of the scorecard for the child group was 8/17 or 47.1%, giving an accuracy for the child group which is also 47.1%.

**Table 2:** Aetiology of sore throat: child group

	Swab		Number of patients
Score Card	Bacterial	Non-bacterial	

Positive	7	9	16
	(True Positive)	(False Positive)	

Negative	0	8	8
	(False Negative)	(True Negative)	

N	7	17	24

### Adult group

In the adult group, 21 patients had a true positive result for bacterial infection, while 2 had a false negative (negative on scorecard but positive on microbiological culture) result (See Table 3). This gives a true positive rate or sensitivity for bacterial infection in the adult group of 21/23 or 91.3%. Similarly, 19 patients had a false positive result for non-bacterial infection while 40 patients had a true negative result. This means that the specificity for the adult group equalled 40/59 or 67.8%. The accuracy of the scorecard for the adult group was therefore 91.3% × 67.8% or 61.9%.

**Table 3:** Aetiology of sore throat: adult group

	Swab		N
Score Card	Bacterial	Viral	

Positive	21	19	40
	(True Positive)	(False Positive)	

Negative	2	40	42
	(False Negative)	(True Negative)	

N	23	59	82

Streptococcal group A was only identified in 11/106 or 10% of the patients with sore throat (Table 4). The scorecard's positive predictive value for bacterial sore throat was 50% in both adult and child groups and the negative predictive value was 96% in both groups. The sensitivity in the adult group was 91.3% as compared with 100% sensitivity in the child group, with an overall sensitivity of 93.3%. The specificity in the adult group was 67.8% as compared with a specificity of 47.1% in the child group, and with an overall specificity of 63.2%. The accuracy of the scorecard was 58.9%. Patient of age less than 16 years was the only criterion out of the ten in the scorecard that emerged as an independent predictor of sore throat streptococcal infection. The combined group bacterial and non-bacterial positive and negative ratios are listed in (Table 5).

**Table 4:** Aetiology of sore throat by group

	Children		Adult		TOTAL
**Aetiology**	**Male**	**Female**	**Male**	**Female**	

Normal throat flora	11	6	19	40	76

Strep A	2	2	3	4	11

Strep B	0	1	1	0	2

Strep C	0	1	3	5	9

Strep G	0	0	2	2	4

Strep pyogenes	0	0	0	1	1

Staph aureus	1	0	2	0	3

Total number of subjects	14	10	30	52	106

**Table 5:** Aetiology of sore throat for all subjects (combined)

	Swab		Number of patients
**Score Card**	**Bacterial**	**Non-bacterial**	

Positive	28	28	56
	(True Positive)	(False Positive)	

Negative	2	48	50
	(False Negative)	(True Negative)	

N	30	76	106

A total of 94.3% of patients agreed that they would rely on their doctor to decide whether or not they needed an antibiotic. If there was a reliable scorecard, 85.8% of patients would trust such a scorecard.

### Validation of the scorecard

The Pearson product-moment correlation between the scorecard and the throat swab for the guardian group was -0.454 (significant at the .05 level), and for the adult group was -0.531 (significant at the .01 level). In general, correlation coefficients of at least 0.7 are considered 'strong', although the above results are acceptable.

Cronbach's alpha for the guardian (i.e. child) group was 0.685 and for the adult group was 0.670, and these are acceptable values for internal reliability.

## Discussion

The sensitivity of the scorecard was 93.3% and the specificity was 63.2%, giving an accuracy of 58.9%. This means that the scorecard gave a reliable answer in just over half (i.e. 58.9%) of patients who presented with a sore throat. Most other scoring systems have either a lower or at least no better accuracy. Although more sensitive, the new scorecard has a lower specificity (63.2%) than other scoring systems, e.g. Breese (85%) and Centor (67%-91%) [2]. The new scorecard is therefore, with a sensitivity of 93.3%, more likely to confirm a bacterial sore throat than other previous scorecards, but a little less likely to identify a 'true negative', which is a viral sore throat. The implication of this in clinical practice is that doctors may be more confident when the scorecard indicates a viral sore throat, but when it predicts a bacterial sore throat, given that the scorecard has a low specificity, (i.e. a high false positive rate) we cannot be absolutely confident that it is bacterial. Follow up of the patient may be required to ensure that the sore throat resolves as would be expected for a viral cause, and if it does not, it should be treated as bacterial.

Apart from age less than 16 years, none of the other ten criteria in the scorecard emerged as an independent predictor of bacterial infection for sore throat.

There are several limitations to this study. First, the current study was conducted by only one investigator with a fairly small number of subjects (106). Hence further studies may be necessary to confirm the findings. Comparable studies had higher numbers of subjects, for example, there were 234 in Centor's study [12], 670 in Breese's [4], 513 in McIsaac's [13] and 418 in Walsh's [14].

No sample size calculation was undertaken to determine how many patients with sore throat are required to develop a more accurate scorecard than existing ones. In that sense, this study must be considered to be a pilot study.

Other researchers have used only bacterial throat swab cultures to investigate sore throats and have not done viral cultures on pragmatic grounds [15,16]. The diagnosis of viral infection is therefore presumptive. The same applies to this study.

The original wording of two of the items in the scorecard could have been clearer. Questions 7 and 10 were stated negatively and were therefore reverse scored in the analysis. They could have been positively stated in the first place and then reversely scored if necessary.

Further, there were limitations with criterion 4 which referred to a history of exposure to a person with bacterial sore throat infection (e.g. GAS, Group C, Strep pyogenes). It is of course hard to prove the cause of a sore throat from the taking of a history. This criterion was often scored as a "No" in doubtful cases (i.e. non-bacterial). This might have skewed the overall scorecard result toward the non-bacterial causes, but the extent of such an effect is unknown. It should be noted that the scorecard cannot differentiate between Streptococcal groups A, B, C or G based on clinical presentation.

The usefulness in patients under 5 years of age is unknown as they were excluded from the study, given that it would have been too difficult to take throat swabs from them.

Although patient trust in the use of the scorecard is high, there is some evidence that where laboratory tests are available and practical, such as the rapid antigen test, patient trust in their doctor's judgement, with or without a scorecard, is diminished [17]. However, as the use of these tests remains severely limited, clinical scorecards such as the new one can be helpful as a practical tool for decision making in general practice.

## Conclusions

The sore throat scorecard presented in the current study appears to be more sensitive than several others published in the literature. However, its specificity is a little less. While all the other scoring systems focused on Streptococcal group A infection only, the new scorecard also included other bacterial organisms as pathogens.

The scorecard could predict non-bacterial sore throat in 96% of cases when the bacterial score was less than or equal to four (i.e. its negative predictive value). However, the positive predictive value of the scorecard was 50%, meaning that the scorecard could predict bacterial sore throat infection in about 50% of the cases when the bacterial score was greater than or equal to five.

Ideally, the scorecard would be both sensitive and specific. However, given that the problem with antibiotic use in general practice tends to be overuse, it would be preferable if the scorecard identified few false positives. Unfortunately, given the relatively low specificity, "positive" results may give more false positives than ideal. On the other hand, a "negative" result is clinically significant in that it tends to rule out bacterial sore throat because the new scorecard has a sufficiently high sensitivity (93.3%), i.e. low false negatives. In summary, the scorecard is most useful when the result is negative.

Further research is required to study the attitudes of doctors to the scorecard at the point of care. However, trust by patients was high.

## Competing interests

The principal author (TB) is the GP of the patients recruited into the study who were given written information about the project and were able to refuse the invitation. Otherwise, the authors declare that they have no competing interests.

## Authors' contributions

TB conceived, designed, and conducted the research and acquired the data for the study at his general practice in rural New South Wales, Australia. TB conducted the analysis and interpretation of data. PS further contributed to the design of the study, the analysis and interpretation of data and its clinical applications. Both TB and PS were involved in drafting the manuscript and revising it critically for important intellectual content and have given final approval of the version to be published.
